# Correlation and predictive value of TLR2, MyD88, and MUC5AC in bronchial mucus plug formation in children with mycoplasma pneumoniae pneumonia: a prospective study

**DOI:** 10.1186/s12890-026-04608-1

**Published:** 2026-08-15

**Authors:** Baowen Li, Peng Shen, Dan Li, Bopei Han, Ling Liu, Tuanjie Wang, Shujun Li

**Affiliations:** 1 Department of PICU, the First Affiliated Hospital of Henan Medical University, Weihui, 453100 China; 2 School of Medicine, Nankai University, Tianjin, 300071 China

**Keywords:** Mycoplasma pneumoniae pneumonia, Bronchial mucus plugs, Toll-like receptor 2, Mucin 5AC, Predictive model

## Abstract

**Background:**

Bronchial mucus plugs (BMPs) are a severe complication of Mycoplasma pneumoniae pneumonia (MPP) in children. This study aimed to investigate the potential mechanisms underlying BMP formation based on biomarkers in bronchoalveolar lavage fluid (BALF) and to develop a predictive model for clinical risk assessment of BMP formation.

**Methods:**

This prospective study enrolled 151 children with MPP hospitalized at the First Affiliated Hospital of Xinxiang Medical University between July 2023 and February 2025. According to the presence of BMPs, patients were classified into BMP (*n* = 66) and non-BMP (*n* = 85) groups. BALF levels of Toll-like receptor 2 (TLR2), myeloid differentiation primary response 88 (MyD88), and mucin 5AC (MUC5AC) were determined via ELISA. Key risk factors identified through LASSO and support vector machine (SVM) were used to construct a multivariate logistic regression-based nomogram, and evaluate its predictive performance. Spearman correlation and serial mediation analyses were employed to explore the TLR2/MyD88/MUC5AC axis in BMP formation.

**Results:**

Compared with the non-mucus plug group, children in the mucus plug group exhibited significantly higher BALF levels of TLR2, MyD88, and MUC5AC (all *P* < 0.001). D-dimer, TLR2, and MUC5AC were identified as independent risk factors for BMP formation. The nomogram demonstrated favorable predictive performance (AUC = 0.914, 95%CI: 0.871–0.958) with good calibration and clinical utility. Significant positive correlations were observed among TLR2, MyD88, and MUC5AC. After adjustment for potential confounders, Serial mediation analysis suggested that the association between TLR2 and bronchial mucus plug formation may involve a serial pathway mediated by MyD88 and MUC5AC.(standardized effect β = 0.026, 95%CI: 0.007–0.052).

**Conclusion:**

In children with MPP, TLR2 may be associated with BMP formation, and this association may partially involve a serial pathway including MyD88 and MUC5AC. D-dimer, TLR2, and MUC5AC are independent risk factors for this condition. The derived nomogram demonstrates high discriminative power and accuracy and may serve as a risk stratification tool during bronchoscopic evaluation to identify children at high risk of extensive mucus plugging and support individualized airway management.

## Introduction

Mycoplasma pneumoniae pneumonia (MPP) is one of the most common forms of community-acquired pneumonia (CAP) in children, accounting for approximately 20%–40% of cases, with epidemic peaks occurring every 3–7 years [1]. In recent years, the incidence of MPP has been increasing, accompanied by a rising proportion of severe and refractory cases [2]. Clinically, some children with MPP develop bronchial mucus plugs (BMPs), which are major contributors to airway obstruction, impaired ventilation, and poor prognosis [3]. Recent studies have reported that BMPs occur in more than 30% of children with severe or refractory MPP [4]. Therefore, understanding the mechanisms underlying mucus plug formation and identifying effective predictive biomarkers are critical for risk stratification and optimization of therapeutic strategies.

Mucin 5AC (MUC5AC) is a major component of airway mucus and can be markedly upregulated under inflammatory stimulation, contributing to mucus hypersecretion [5]. Mycoplasma pneumoniae infection can induce MUC5AC secretion by airway epithelial goblet cells, increasing mucus viscosity and airway obstruction [6]. Toll-like receptor 2 (TLR2), an important pattern recognition receptor, recognizes Mycoplasma pneumoniae-associated molecular patterns and activates innate immune responses [7]. Myeloid differentiation primary response 88(MyD88) is a key adaptor molecule in Toll-like receptor (TLR)-mediated signaling pathways. The TLR2/MyD88 signaling pathway serves as an important regulatory axis involved in the development and progression of various diseases and participates in pathogen recognition, inflammatory responses, and tissue injury processes [8].Yujie Fan et al. demonstrated that TLR2 and MyD88 expression levels were significantly elevated in bronchoalveolar lavage fluid (BALF) from children with MPP and positively correlated with High-mobility group box protein 1༈HMGB1༉, suggesting that the TLR2/MyD88 signaling pathway may contribute to local pulmonary immune activation following MP infection [9]. In addition, experimental studies have shown that activation of the TLR/MyD88 signaling axis can regulate airway MUC5AC expression under inflammatory conditions, indicating its potential involvement in airway mucus hypersecretion [10]. However, clinical evidence linking the TLR2/MyD88 signaling pathway to bronchial mucus plug formation in children with MPP remains limited, with most current studies focusing predominantly on peripheral serological markers that may not fully reflect localized microenvironmental changes in the lungs. Meanwhile, existing predictive models for pediatric MPP have mainly focused on disease severity assessment using routine clinical features and radiological findings, with limited attention to airway-derived biomarkers and BMP-specific prediction. BALF, obtained from affected lung segments via flexible bronchoscopy, provides a more direct representation of pulmonary inflammatory cell infiltration and localized secretion accumulation.Therefore, integrating BALF-derived innate immune and mucus-related biomarkers into predictive models may provide additional mechanistic insights and improve the early identification of children at high risk for bronchial mucus plug formation.

However, traditional logistic regression may be susceptible to multicollinearity and overfitting when analyzing multiple clinical parameters. Machine learning algorithms, such as least absolute shrinkage and selection operator (LASSO) regression and support vector machines (SVM), excel at feature selection and pattern recognition in complex datasets. Applying LASSO regression and SVM algorithms provides complementary approaches for feature selection, helping to reduce multicollinearity and improve model generalizability [11, 12].

Based on these considerations, the present study aimed to investigate the associations between BALF levels of MUC5AC, TLR2, and MyD88 and the formation of bronchial mucus plugs in children with MPP. Candidate predictors were first screened independently using LASSO regression and SVM algorithms, followed by multivariate logistic regression to identify independent risk factors. A predictive nomogram was then constructed based on these independent factors, and its performance was evaluated, with the goal of identifying children at high risk of extensive downstream mucus plugging and supporting individualized airway management strategies.

## Methods

### Study population and grouping

This prospective study enrolled children with MPP who were hospitalized in the pediatric ward of the First Affiliated Hospital of Xinxiang Medical University between July 2023 and February 2025. The inclusion criteria were as follows: age ≤ 14 years; met the diagnostic criteria for MPP as defined in the Evidence-based Guideline for the Diagnosis and Treatment of Mycoplasma pneumoniae Pneumonia in Children (2023 edition) [13]; meeting the indications for flexible bronchoscopy according to the Guideline of Pediatric Flexible Bronchoscopy in China (2018 version) [14]; and provision of written informed consent by guardians. The exclusion criteria were as follows: incomplete clinical data; coexistence of chronic pulmonary diseases, immune disorders, congenital airway developmental abnormalities, bronchopulmonary malformations, inherited metabolic diseases, or congenital cardiopulmonary diseases; and co-infection with other pathogens. A total of 208 children were initially screened, of whom 57 were excluded, including 24 who did not undergo bronchoscopy with bronchoalveolar lavage, 19 with incomplete medical records, 9 with other pathogen infections confirmed by etiological testing within 1 week after admission, 3 with congenital cardiopulmonary diseases, 1 with severe malnutrition, and 1 with an inherited metabolic disease. Ultimately, 151 eligible children with MPP were included in this prospective cohort study (76 males and 75 females), and BALF samples were collected for analysis. According to the presence or absence of bronchial mucus plugs identified by bronchoscopy, patients were divided into the BMP group (*n* = 66) and the non-BMP group (*n* = 85). The sample size for this study was justified based on the events per variable (EPV) principle in logistic regression analysis. To ensure the stability and reliability of the multivariate logistic regression model, an EPV of ≥ 5–10 is generally recommended. After clinical evaluation and statistical screening, five core predictive variables, including TLR2, D-dimer, MUC5AC, MyD88, and lactate dehydrogenase(LDH), were identified and subsequently included in the multivariate logistic regression analysis. Therefore, at least 25–50 outcome events were theoretically required for model development. In this study, 66 patients with bronchial mucus plug formation were included, resulting in an EPV of 13.2 (66/5), which exceeded the recommended threshold and supported the adequacy of the sample size for model construction. This study was approved by the Medical Ethics Committee of the First Affiliated Hospital of Xinxiang Medical University (Ethics No. Ec-023-155), and written informed consent was obtained from the guardians of all participants.

### BALF collection and analysis

In children with MPP, bronchoalveolar lavage was performed in the lung segment or lobe with the most severe lesions as identified by radiological and bronchoscopic findings.All children underwent electronic bronchoscopy and bronchoalveolar lavage within 24–48 h after admission. The bronchoalveolar lavage procedure was performed in accordance with the Guideline of Pediatric Flexible Bronchoscopy in China (2018 version) [13].Sterile normal saline was used for bronchoalveolar lavage at a volume of 1mL/kg, with a total volume not exceeding 20 mL. A recovery rate of ≥ 40% was considered an acceptable specimen. All BALF specimens were immediately centrifuged at 1000 rpm for 10 min at 4 °C after collection. The supernatants were subsequently harvested and stored at − 80 °C until analysis.To evaluate the levels of key innate immune-related biomarkers, the concentrations of MUC5AC, soluble TLR2 (sTLR2), and MyD88 in BALF supernatants were measured using enzyme-linked immunosorbent assay (ELISA). Commercial ELISA kits designed for human MUC5AC (EH1035, detection range: 0.313–20 ng/mL, lower limit of detection [LLOD]: 0.188 ng/mL; Fine Biotech Co., Ltd., Wuhan, China), TLR2 (EH0300, detection range: 0.156–10 ng/mL, LLOD: 0.094 ng/mL; Fine Biotech Co., Ltd.), and MyD88 (EH0240, detection range: 0.156–10 ng/mL, LLOD: 0.094 ng/mL; Fine Biotech Co., Ltd.) were utilized according to the manufacturer’s instructions. Samples were appropriately diluted before analysis when necessary to ensure that the measured concentrations fell within the standard curve range. The final concentrations were calculated by multiplying the measured values by the corresponding dilution factors. According to the manufacturer’s validation data, these kits demonstrated acceptable spike-recovery (range: 85%–105%) and precision, with intra-assay and inter-assay coefficients of variation (CV) strictly below 7%. All measurements were performed in duplicate, and values below the LLOD were excluded from analysis. All laboratory tests, including ELISA measurements of BALF TLR2, MyD88 and MUC5AC, were performed by technicians who were blinded to the patients’ clinical grouping (BMP vs. non-BMP).The median interval from symptom onset (fever or cough) to BALF collection was 4 days (IQR: 3–6 days) in the non-BMP group and 6 days (IQR: 4–7 days) in the BMP group (*P* = 0.059). The median interval from hospital admission to bronchoscopy was 1 day (IQR: 1–2 days) in the non-BMP group and 2 days (IQR: 1–2 days) in the BMP group (*P* = 0.070). The median interval from antibiotic initiation to bronchoscopy was 3 days (IQR: 2–4 days) in both the non-BMP and BMP groups (*P* = 0.166).

### Clinical data collection

Clinical data of all enrolled children with MPP were collected from the electronic medical record system, including: Demographic characteristics (e.g., sex and age). Clinical course data (length of hospital stay, duration of fever, and duration of fever after antibiotic treatment). Clinical manifestations (peak temperature, dyspnea, and chest pain). Laboratory test results (e.g., C-reactive protein [CRP], D-dimer, and complement C4 levels). Radiological features (pleural effusion, pericardial effusion, and consolidation involving ≥ 2/3 of the lung lobes). Treatment information (the use of second-line antibiotics and intravenous immunoglobulin [IVIG]). In addition, macrolide resistance-associated gene mutation (23 S rRNA-A2063G) status was recorded. All laboratory examinations were completed within 24 h after admission.Clinical data and outcome assessments were recorded by attending physicians who were not aware of the BALF biomarker results at the time of data entry.

### Statistical analyses

Statistical analyses were performed using SPSS version 26.0 and R version 4.2.3 The normality of continuous variables was assessed using the Shapiro–Wilk test. Non-normally distributed data were expressed as median (interquartile range) [M (P25, P75)], and comparisons between groups were performed using the Mann–Whitney U test. Categorical variables were expressed as *n* (%) and compared using the chi-square test. For the analysis of clinical characteristics, *P* values were adjusted for multiple comparisons using the Benjamini–Hochberg false discovery rate (FDR) procedure. Spearman correlation analysis was used to evaluate correlations among continuous variables. LASSO regression and SVM algorithms were applied to identify candidate predictors of bronchial mucus plug formation, and the selected variables were subsequently included in multivariate logistic regression analysis to construct the predictive model. For variables with rare events and potential estimation instability, Firth’s penalized logistic regression was additionally performed using R version 4.2.3 as a sensitivity analysis. Receiver operating characteristic (ROC) curve analysis was performed to assess the discriminative ability of the model, while calibration curves and decision curve analysis (DCA) were used to evaluate the calibration performance and clinical utility of the model.Internal validation was performed using bootstrap resampling with 1,000 repetitions to assess model stability and optimism-corrected performance. An exploratory serial mediation analysis was performed using structural equation modeling (SEM) in R version 4.2.3, adjusting for potential confounders. 95% confidence intervals (CIs) excluding zero indicated statistical significance. A two-sided *P* < 0.05 was considered statistically significant.

## Results

### Clinical characteristics of children in the non-BMP and BMP groups

We compared the clinical characteristics of children with MPP between the BMP group and the non-BMP group. No significant differences were observed between the two groups in sex, age, height, platelet count, creatine kinase-MB (CK-MB), uric acid, creatinine, activated partial thromboplastin time (APTT), prothrombin activity (PTA), thrombin time (TT), international normalized ratio (INR), or macrolide resistance mutation (23 S rRNA-A2063G) after correction for multiple comparisons (all *P_adj* > 0.05). Children in the mucus plug group had longer hospital stays, longer fever durations, and prolonged fever after antibiotic treatment. Furthermore, neutrophil count, CRP, D-dimer, alanine aminotransferase(ALT), and LDH levels were higher, whereas albumin and complement C4 levels were lower in the BMP group (all *P_adj* < 0.05). The BALF levels of TLR2, MyD88, and MUC5AC were significantly elevated in children with MPP in the BMP group compared with those in the non-BMP group (all *P_adj* < 0.001). Regarding clinical and radiological manifestations, the mucus plug group showed higher peak temperatures and increased incidences of dyspnea and pleural effusion (all *P_adj* < 0.05). Although consolidation involving ≥ 2/3 of the lung lobes was more frequent in the BMP group, this difference did not remain significant after FDR correction. In terms of treatment-related variables, IVIG administration and mechanical ventilation were more common in the BMP group, whereas the difference in second-line antibiotic use did not remain statistically significant after adjustment (Table 1).

**Table 1:** Clinical characteristics of children in the non-BMP and BMP groups

Variable	The non–BMP group (*n* = 85)	The BMP group(*n* = 66)	Z/χ^2^	*P*	P_adj
Male, n (%)	48(56.5)	28(42.4)	2.932	0.087	0.172
Height(cm)	125.0(110.0, 135.8)	125.0(115.0, 137.3)	-0.831	0.406	0.488
Age (years)	7.0(4.0, 8.0)	7.0(5.0, 9.0)	0.743	0.295	0.519
Length of hospital stay (days)	8.0(7.0, 10.0)	10.0(8.0, 13.0)	3.474	< 0.001	0.002
Duration of fever (days)	6.0(5.0, 9.0)	8.0(5.3, 11.0)	2.852	0.004	0.010
Duration of fever after antibiotic treatment (days)	1.0(0.0, 2.0)	2.0(1.0, 4.0)	2.797	0.005	0.012
Laboratory parameters					
Neutrophil count(×10^9^/L)	5.2(4.0, 7.2)	6.1(4.9, 8.6)	2.513	0.012	0.024
Platelet count(×10^9^/L)	286.0(240.0, 369.0)	257.0(226.0, 351.3)	-1.030	0.303	0.380
Lymphocyte count(×10^9^/L)	1.9(1.2, 2.6)	1.5(1.0, 2.7)	-1.748	0.080	0.128
CRP(mg/L)	15.6(7.8, 37.3)	32.9(16.7, 82.5)	3.997	< 0.001	< 0.001
APTT(sec)	34.9(30.7, 40.1)	33.1(29.6, 38.5)	-1.559	0.119	0.172
D-dimer(ug/ml)	0.8(0.6, 1.1)	2.3(0.9, 4.6)	-5.723	< 0.001	< 0.001
PTA(%)	78.3(73.0, 86.2)	77.8(72.2, 87.1)	-0.263	0.793	0.842
TT(sec)	14.8(14.3, 15.4)	14.7(14.1, 15.8)	-0.362	0.717	0.786
INR	1.1(1.0, 1.1)	1.1(1.0, 1.1)	-0.811	0.417	0.488
ALT(U/L)	14.0(11.0, 19.0)	19.5(13.3, 29.0)	3.364	< 0.001	0.002
LDH(U/L)	257.0(231.0, 317.0)	379.0(278.5, 554.0)	4.516	< 0.001	< 0.001
Albumin(g/L)	41.3(39.1, 42.9)	38.5(36.2, 41.4)	-3.754	< 0.001	< 0.001
CK-MB(ng/ml)	2.1(1.4, 3.0)	2.4(1.6, 3.5)	-1.546	0.122	0.172
Complement C4(g/L)	0.3(0.3, 0.5)	0.3(0.2, 0.4)	-3.133	0.002	0.005
Uric acid(umol/L)	3.3(2.6, 4.1)	3.1(2.7, 4.1)	-0.124	0.901	0.927
Creatinine(umol/L)	32.2(25.3, 40.1)	31.2(25.2, 41.6)	-0.094	0.925	0.927
TLR2(ng/mL)	2.0(1.0, 4.4)	17.9(11.9, 32.6)	8.988	< 0.001	< 0.001
MyD88(ng/mL)	6.0(2.5, 14.8)	17.4(11.2, 24.0)	5.042	< 0.001	< 0.001
MUC5AC(ng/mL)	9.0(2.0, 14.9)	18.3(13.2, 26.0)	6.383	< 0.001	< 0.001
Clinical manifestations					
Peak temperature (°C)	39.3(39.0, 39.7)	39.7(39.2, 40.0)	3.452	< 0.001	0.002
Chest pain, n(%)^*^	0	4(6.1)	3.202	0.074	0.061
Dyspnea, n(%)^*^	1(1.2)	10(15.1)	8.774	0.003	0.003
Radiological findings					
Pleural effusion, n(%)	11(12.9)	26(39.4)	14.053	< 0.001	0.002
Consolidation involving ≥ 2/3 of the lung lobes, n(%)	63(74.1)	58(87.9)	4.419	0.036	0.096
Pericardial effusion, n(%)	5(5.9)	8(12.1)	1.838	0.175	0.373
Interventions					
Use of second-line antibiotics, n(%)	39(45.9)	43(65.1)	5.559	0.018	0.052
Use of intravenous immunoglobulin (IVIG), n(%)^#^	0	7(10.6)	-	0.003	0.006
Mechanical ventilation, n(%)^#^	0	8(12.1)	8.598	0.003	0.022
Macrolide resistance mutation (23 S rRNA-A2063G)	12(14.1)	15(22.7)	1.876	0.171	0.334

*P* < 0.05 was considered statistically significant

Data are presented as median (interquartile range, IQR) or *n* (%);* indicates that the chi-square test with continuity correction was used; # indicates that Fisher’s exact test was applied;*P_adj* were calculated using the Benjamini–Hochberg false discovery rate (FDR) procedure to account for multiple comparisons

### Univariate logistic regression analysis of risk factors for BMP formation in children with MPP

Based on the results of Table 1 and considering clinical relevance, variables potentially associated with BMP formation were selected as candidate predictors and subsequently included in the univariate logistic regression analysis. Variables with extremely low event frequencies were not included in the analysis. The results showed that neutrophil count, CRP, D-dimer, LDH, TLR2, MyD88, MUC5AC, peak temperature, dyspnea, pleural effusion, and consolidation involving ≥ 2/3 of the lung lobes were identified as risk factors for bronchial mucus plug formation (*OR* > 1), whereas albumin, and complement C4 were identified as protective factors (*OR* < 1). In addition, ALT were not statistically significant (*P* > 0.05) (Table 2).

**Table 2:** Univariate logistic regression analysis of risk factors for BMP formation

Variable	B	SE	Wald	*P*	OR	95%CI
Neutrophil count	0.139	0.059	5.476	0.019	1.149	(1.023, 1.291)
CRP	0.018	0.005	12.511	<0.001	1.018	(1.008, 1.028)
D-dimer	0.646	0.148	19.062	<0.001	1.909	(1.428, 2.552)
ALT	0.014	0.008	2.840	0.092	1.014	(0.998, 1.030)
LDH	0.005	0.001	16.714	<0.001	1.005	(1.003, 1.008)
Albumin	-0.143	0.047	9.077	0.003	0.867	(0.790, 0.951)
Complement C4	-2.962	1.086	7.447	0.006	0.052	(0.006, 0.434)
TLR2	0.085	0.021	16.853	<0.001	1.089	(1.045, 1.134)
MyD88	0.083	0.019	20.095	<0.001	1.087	(1.048, 1.127)
MUC5AC	0.109	0.023	22.792	<0.001	1.115	(1.066, 1.166)
Peak temperature	1.017	0.303	11.289	0.001	2.764	(1.528, 5.002)
Dyspnea^a^	2.348	0.890	6.968	0.001	10.469	(2.358, 98.913)
Pleural effusion	1.475	0.410	12.966	<0.001	4.373	(2.003, 10.096)
Consolidation involving ≥ 2/3 of the lung lobes	0.929	0.451	4.239	0.040	2.532	(1.081, 6.468)

ᵃ Firth’s penalized logistic regression was applied for variables with rare events

### LASSO regression and SVM analyses for BMP formation in children with MPP

**Fig. 1:**
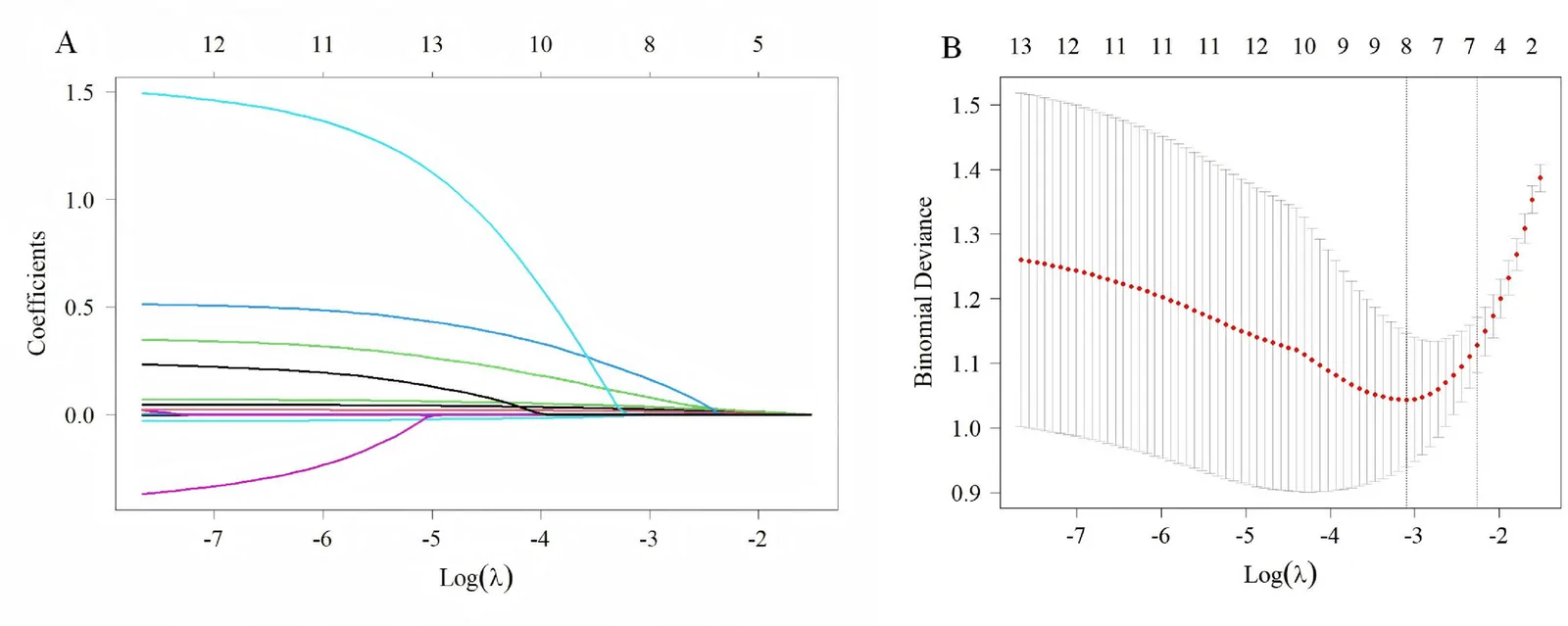
LASSO regression analysis for predictor selection of BMP formation in children with MPP. **A** LASSO coefficient profile plot. **B** Cross-validation plot for selection of the optimal penalty parameter

**Fig. 2:**
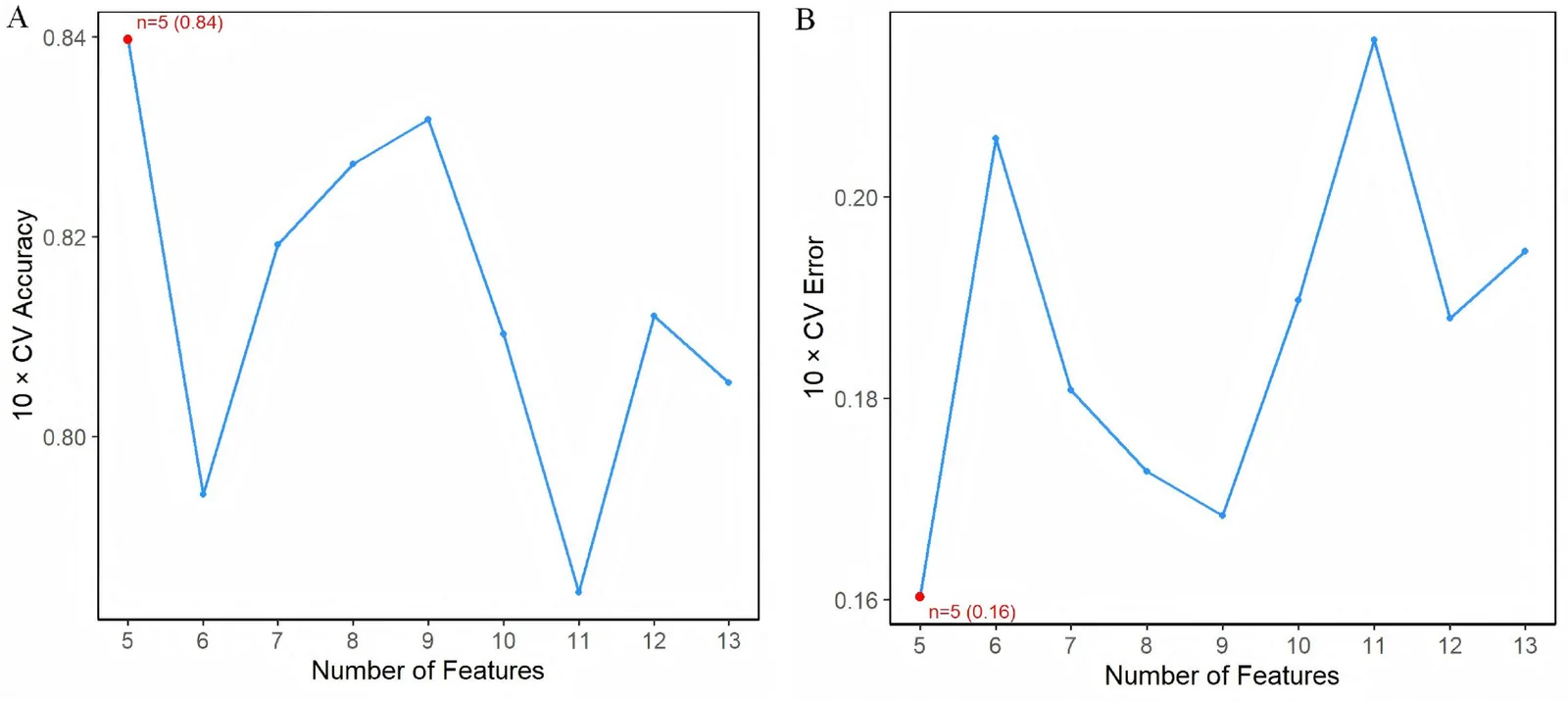
Feature selection using the SVM model for predicting BMP formation in children with MPP. **A** Relationship between the number of features and cross-validated accuracy. **B** Relationship between the number of features and cross-validated error

To identify key predictors associated with bronchial mucus plug formation in children with MPP, variables showing significant associations in univariate logistic regression analysis (*P* < 0.05) were independently subjected to LASSO regression and SVM analysis for feature selection. In the LASSO regression analysis, the changes in regression coefficients and partial likelihood deviance with log10(λ) are presented in Fig. 1, respectively. Based on the optimal λ value determined by cross-validation, a total of eight variables with nonzero coefficients were identified, including CRP, D-dimer, LDH, TLR2, MyD88, MUC5AC, peak temperature, and dyspnea. Subsequently, SVM analysis with 10-fold cross-validation was performed (Fig. 2). The results demonstrated that the model achieved optimal predictive performance when five feature variables were included, yielding an accuracy of 0.84 and an error rate of 0.16. The five selected variables were TLR2, D-dimer, MUC5AC, MyD88, and LDH. Further increasing the number of features did not improve model accuracy and slightly increased the error rate, suggesting that these five variables achieved the best balance between predictive performance and model complexity. The intersection of variables selected by LASSO regression and SVM analyses was considered as stable candidate predictors. Ultimately, TLR2, D-dimer, MUC5AC, MyD88, and LDH were identified as the core predictive variables and were subsequently included in the multivariate logistic regression analysis.

### Multivariate logistic regression analysis of BMP formation in MPP

Variables identified by both the LASSO regression and SVM analyses were further included as independent variables, with bronchial mucus plug formation in children with MPP as the dependent variable, for multivariate logistic regression analysis using a stepwise regression method. The results demonstrated that D-dimer, TLR2, and MUC5AC levels were independent risk factors for bronchial mucus plug formation in children with MPP (all *P* < 0.05) (Table 3).

**Table 3:** Multivariate logistic regression analysis of BMP formation in pediatric patients with MPP

Variable	B	SE	Wald	*P*	OR	95%CI
D-dimer	0.420	0.191	4.828	0.028	1.523	(1.046, 2.215)
TLR2	0.046	0.015	9.526	0.002	1.047	(1.017, 1.078)
MUC5AC	0.074	0.026	7.978	0.005	1.077	(1.023, 1.134)

### Construction and evaluation of the predictive model

**Fig. 3:**
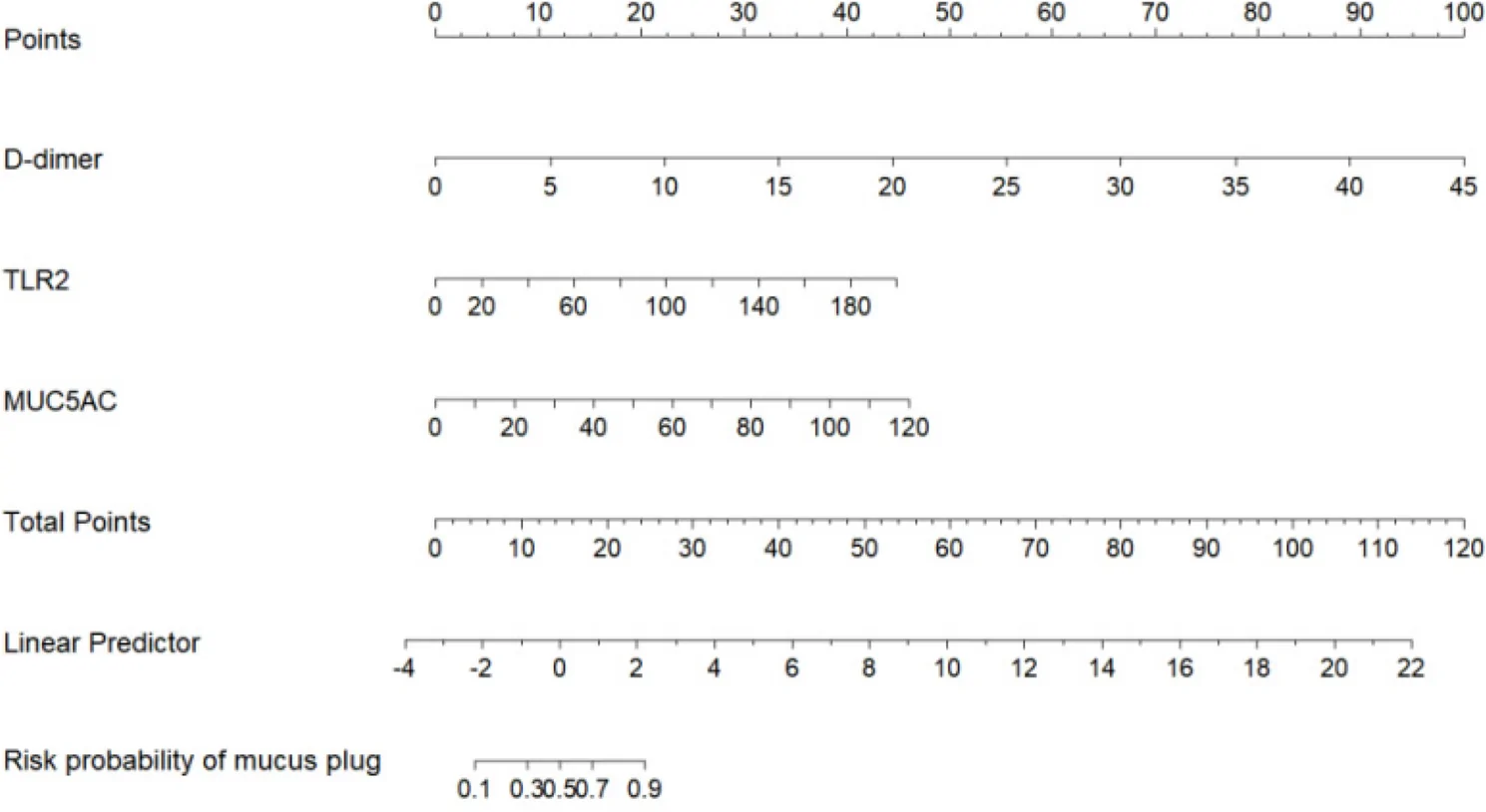
Nomogram for predicting the risk of BMP formation in children with MPP

**Fig. 4:**
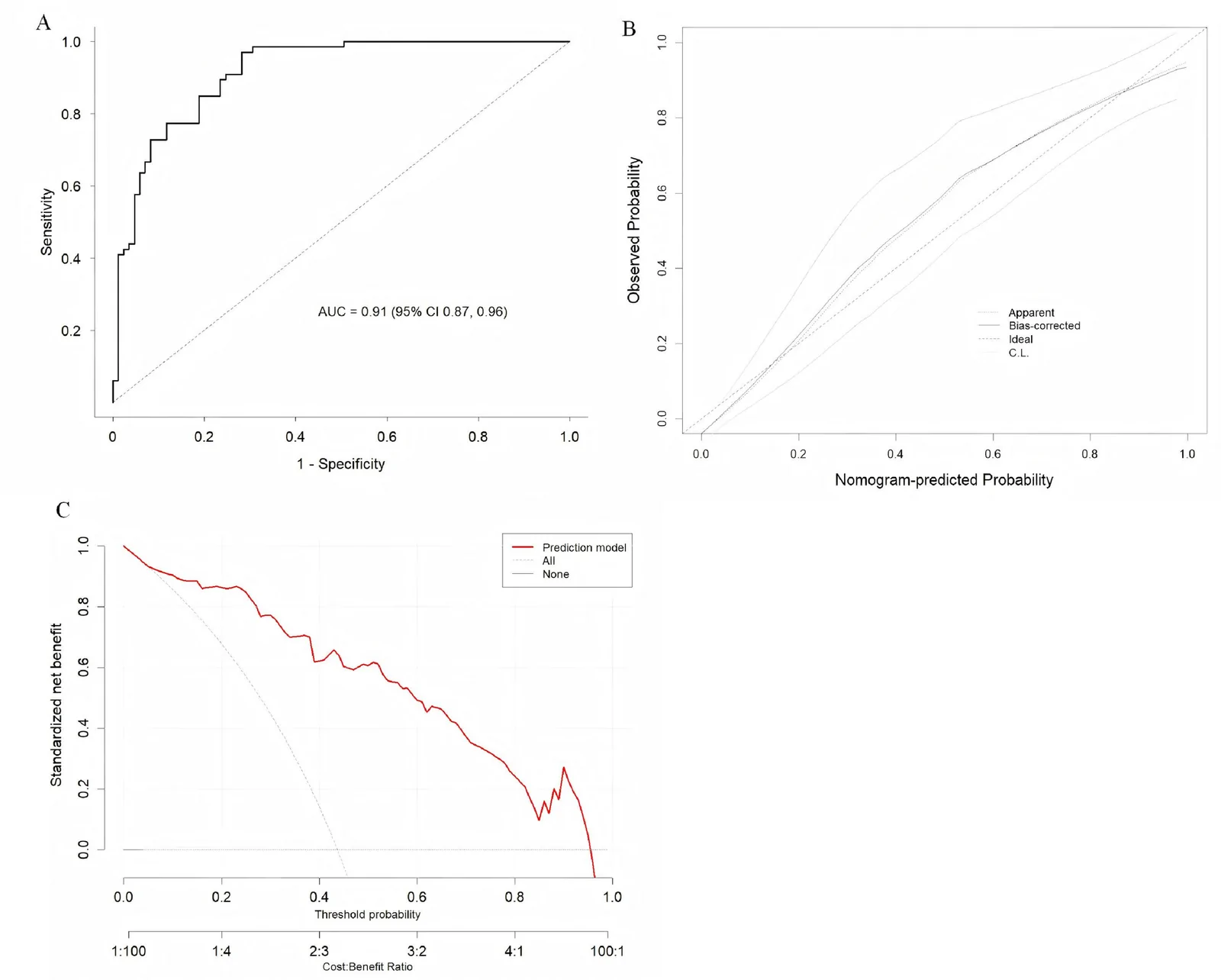
Performance evaluation of the nomogram model for predicting BMP formation in children with MPP. **A** Receiver operating characteristic (ROC) curve analysis. **B** Calibration curve analysis. **C** Decision curve analysis (DCA) of the nomogram model

Based on the results of LASSO regression and SVM-based feature selection followed by multivariable logistic regression analysis, D-dimer, TLR2, and MUC5AC were incorporated to construct a nomogram model for predicting the risk of bronchial mucus plug formation in children with MPP (Fig. 3).ROC curve analysis demonstrated that the model exhibited good discriminative performance, with an AUC of 0.914 (95% CI: 0.871–0.958), a sensitivity of 0.970, a specificity of 0.718, a Youden index of 0.688, and a C-index of 0.914 (Fig. 4A).After 1,000 bootstrap resampling, the optimism-corrected AUC remained 0.908,indicating satisfactory internal validation performance of the model.Calibration analysis based on 1,000 bootstrap resamples demonstrated good agreement between the predicted and observed outcomes. The optimism-corrected Brier score was 0.132, with a calibration intercept of − 0.078 and a calibration slope of 0.861, indicating favorable calibration performance of the model(Fig. 4B). Furthermore, DCA demonstrated that the nomogram provided greater net clinical benefit than the treat-all and treat-none strategies across threshold probabilities ranging from 0.06 to 0.95 (Fig. 4C).

### Correlation analysis between TLR2, MyD88, and MUC5AC in BALF and serum laboratory parameters

Spearman correlation analysis demonstrated that TLR2, MyD88, and MUC5AC levels in BALF were significantly positively correlated with each other (*r* = 0.488–0.585, all *P* < 0.001). In addition, all three markers showed positive correlations with D-dimer and LDH levels (all *P* < 0.05), indicating close associations with coagulation activation and tissue injury. TLR2 and MyD88 were positively correlated with CRP levels, whereas no significant correlation was observed between MUC5AC and CRP (*P* = 0.051). Furthermore, TLR2, MyD88, and MUC5AC were all negatively correlated with Albumin levels (all *P* < 0.05) (Table 4).

**Table 4:** Correlation analysis between TLR2, MyD88, and MUC5AC in BALF and serum laboratory parameters

Variable	TLR2	MyD88	MUC5AC
	*r* (*P*)	*r* (*P*)	*r* (*P*)
TLR2	-	-	-
MyD88	0.500(<0.001)	-	-
MUC5AC	0.488(<0.001)	0.585(<0.001)	-
CRP	0.274(0.001)	0.170(0.037)	0.159(0.051)
D-dimer	0.390(<0.001)	0.277(0.001)	0.270(0.001)
ALT	0.280(<0.001)	0.144(0.078)	0.316(<0.001)
LDH	0.363(<0.001)	0.185(0.023)	0.258(0.001)
Albumin	-0.324(<0.001)	-0.171(0.035)	-0.199(0.014)
Complement C4	-0.197(0.015)	-0.153(0.061)	-0.312(<0.001)

### Serial mediation analysis of TLR2, MyD88, and MUC5AC in BMP formation

**Fig. 5:**
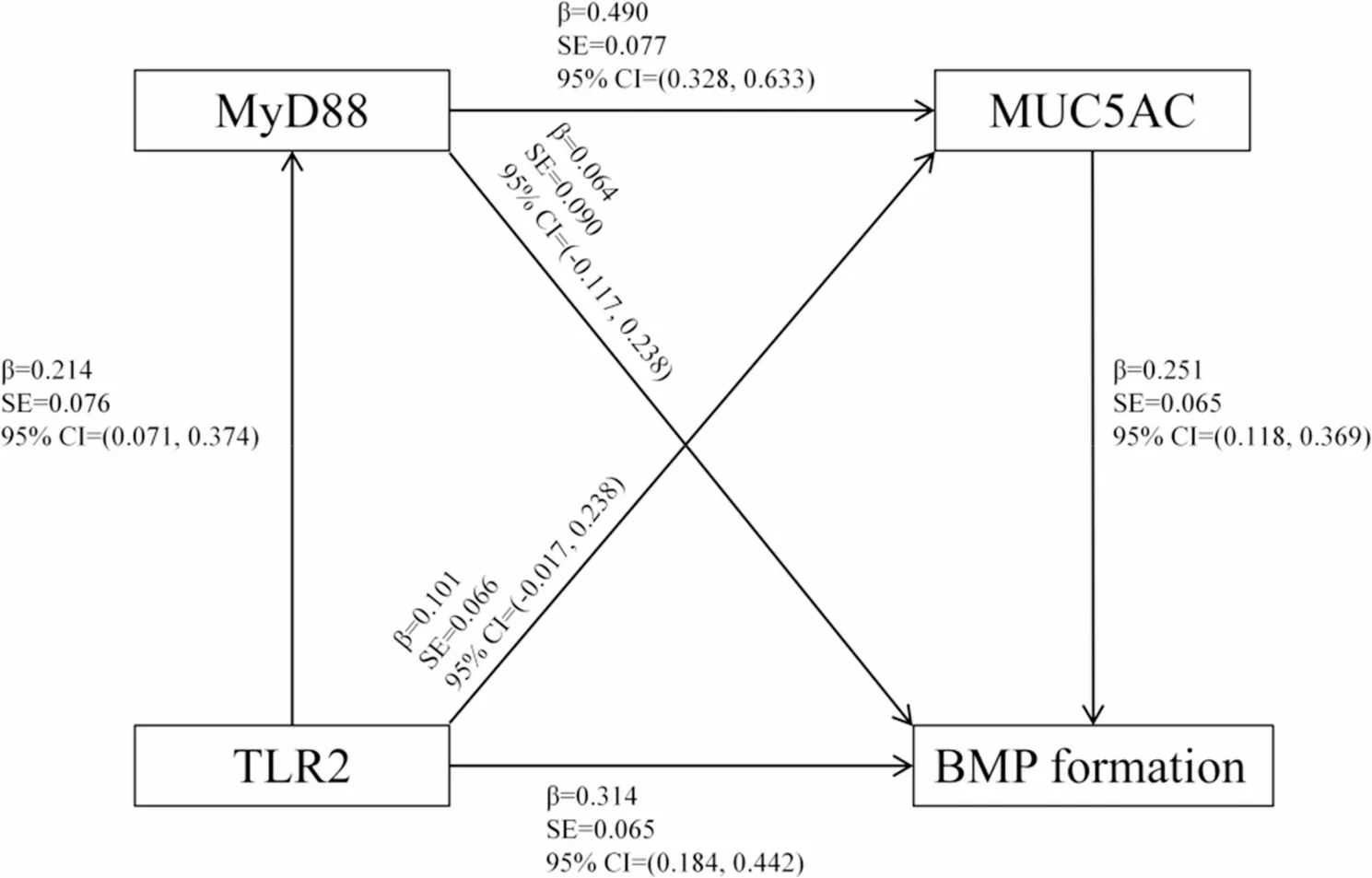
Serial mediation model of TLR2, MyD88, and MUC5AC on BMP formation. Standardized path coefficients (β), standard errors (SE), and confidence intervals (CI) are presented for each pathway

After adjustment for potential confounders, including age, CRP, LDH, and consolidation involving ≥ 2/3 of the lung lobes, serial mediation analysis was performed with BMP formation (binary outcome variable) as the dependent variable. The results showed that TLR2 was significantly associated with BMP formation (standardized total effect β = 0.379, 95% CI: 0.254–0.500), with the direct effect accounting for the majority of the total effect (β = 0.314, 82.94%). Meanwhile, the total indirect effect involving MyD88 and MUC5AC was also statistically significant (β = 0.065, 95% CI: 0.019–0.118). Further analysis demonstrated a significant serial association pathway of TLR2→MyD88→MUC5AC→bronchial mucus plug formation (β = 0.026, 95% CI: 0.007–0.052), suggesting that TLR2, MyD88, and MUC5AC may participate in a serial association pattern related to bronchial mucus plug formation in children with MPP (Table 5; Fig. 5).

**Table 5:** Serial mediation analysis of TLR2, MyD88, and MUC5AC in BMP formation

Pathway	Standardized effect(β)	Standard error(SE)	95%CI	Relative mediation effect (%)
Total effect	0.379	0.063	(0.254,0.500)	100.00
Direct effect	0.314	0.065	(0.184,0.442)	82.94
Total indirect effect	0.065	0.025	(0.019,0.118)	17.06
TLR2→MyD88→BMP formation	0.013	0.021	(-0.027,0.059)	3.35
TLR2→MUC5AC→BMP formation	0.026	0.017	(-0.005,0.061)	6.83
TLR2→MyD88→MUC5AC→BMP formation	0.026	0.012	(0.007,0.052)	6.88

Relative mediation effect (%) = indirect effect / total effect. An effect was considered statistically significant when the 95% confidence interval (CI) did not include zero

## Discussion

This study demonstrated that children with BMP formation exhibited significantly higher BALF levels of TLR2, MyD88, and MUC5AC compared with those without BMPs. Multivariate logistic regression analysis identified D-dimer, TLR2, and MUC5AC as independent risk factors for BMP formation, and the nomogram model constructed based on these indicators exhibited favorable predictive performance and potential clinical utility. In addition, correlation and serial mediation analyses further suggested that the association between TLR2 and BMP formation may be partially explained by a serial pathway involving MyD88 and MUC5AC.

In recent years, with the increasing incidence of severe and refractory MPP in children, early identification and intervention of severe airway injury have become major clinical concerns. BMPs are considered an important factor affecting pulmonary function recovery and prognosis in children with MPP [15–17]. In this prospective study, we found that children in the mucus plug group exhibited longer fever duration, prolonged hospitalization, and higher incidences of pleural effusion and extensive pulmonary consolidation, suggesting that BMP formation is closely associated with more severe airway and systemic inflammatory injury. Notably, levels of D-dimer, and LDH were significantly elevated in the mucus plug group, and D-dimer was further identified as an independent risk factor for BMP formation, which is consistent with previous findings in refractory MPP [18].Increasing evidence suggests that coagulation abnormalities in children with MPP may be closely associated with MP-induced inflammation–coagulation interactions. MP infection can directly or indirectly activate both the coagulation and immune systems, leading to vascular endothelial injury, local inflammatory responses, and microthrombus formation [19]. As the disease progresses, persistent fever, hypoxia, and dehydration may further stimulate immune cells to release pro-inflammatory mediators such as IL-6 and TNF-α, thereby aggravating endothelial injury and contributing to elevated D-dimer levels [20, 21]. Meanwhile, under the influence of these inflammatory and coagulation-related factors, fibrinogen is continuously converted into fibrin deposits. Through factor XIIIa-mediated crosslinking, extensive fibrin networks further aggregate with airway mucus and inflammatory cellular components, ultimately promoting the formation of bronchial mucus plugs [22, 23].

In the present study, MUC5AC levels were significantly elevated in the BMP group compared with the non-BMP group. As an important component of bronchial mucus plugs, MUC5AC plays a critical role in airway mucus hypersecretion [24]. Under pathological conditions, disruption of airway mucus homeostasis may result in abnormal MUC5AC overexpression [25, 26]. MUC5AC-dominant alterations in mucus composition may enhance intermolecular disulfide cross-linking and reduce network pore size, transforming mucus into a highly viscoelastic gel. These changes may substantially impair mucociliary clearance and accelerate mucus retention and plug accumulation [27]. In our study, multivariable logistic regression analysis further identified MUC5AC as an independent risk factor for bronchial mucus plug formation, supporting its potential role in airway obstruction among children with MPP. Further correlation analysis demonstrated significant positive correlations among TLR2, MyD88, and MUC5AC (*r* = 0.488–0.585, all *P* < 0.001), indicating that these molecules may collectively participate in airway mucus hypersecretion in MPP. More importantly, adjusted serial mediation analysis suggested that the association between TLR2 and bronchial mucus plug formation may involve a serial pathway involving MyD88 and MUC5AC (standardized effect β = 0.026, 95% CI: 0.007–0.052), whereas the indirect associations through MyD88 alone or MUC5AC alone did not reach statistical significance. These findings suggest that, compared with the isolated association of individual molecules, the coordinated involvement of the TLR2/MyD88/MUC5AC signaling axis may represent a potential pathway associated with airway mucus hypersecretion in children with MPP. Previous studies have demonstrated that MP can induce the release of pro-inflammatory cytokines, including Interleukin-6(IL-6) and Tumor necrosis factor-alpha(TNF-α), through activation of the TLR2-dependent MyD88/Nuclear factor kappa-B(NF-κB) signaling pathway [28, 29]. Based on our findings, we hypothesize that MP infection may involve dysregulation of the TLR2/MyD88/MUC5AC signaling axis, which is associated with increased production of highly adhesive mucus and aggregation of inflammatory exudates and exfoliated epithelial cells, ultimately leading to bronchial mucus plug formation. These abnormal mucus plugs may further contribute to airway obstruction, atelectasis, and severe ventilatory dysfunction in children with severe MPP [17, 30, 31].

To improve the risk assessment of extensive bronchial mucus plug formation in children with MPP, we constructed a nomogram prediction model based on D-dimer, TLR2, and MUC5AC. Compared with previous models that primarily focused on conventional inflammatory indicators [32, 33], the present model integrated coagulation-related markers with airway mucus secretion–associated indicators, thereby providing a more comprehensive reflection of the pathophysiological mechanisms underlying mucus plug formation. The model demonstrated good apparent discrimination, with an apparent AUC of 0.914 (95% CI: 0.871–0.958). After optimism correction using bootstrap internal validation, the optimism-corrected AUC remained 0.908, indicating satisfactory internal validation performance. In addition, both the calibration curve and DCA confirmed good predictive consistency and potential clinical utility. During variable selection, LASSO regression and SVM algorithms were jointly applied for feature reduction, which helped reduce the risk of overfitting and improve model robustness. Notably, although our results suggest that MyD88 is involved in the “TLR2→MyD88→MUC5AC” serial mediation axis, it was not retained in the final multivariable model. This indicates that MyD88 may primarily exert an intermediary pathway role in BMP formation rather than possessing independent predictive value. Although BALF biomarkers require bronchoscopic collection, this predictive model may serve as a valuable risk stratification tool during initial diagnostic airway evaluation to identify children at high risk of extensive downstream mucus plugging, thereby providing supportive evidence for early intensive airway lavage and mucus-targeted therapy.

Furthermore, recent advances in radiologic segmentation and computer-assisted detection (e.g., CT-based quantification of mucus plugs in cystic fibrosis [34]) have underscored the pivotal clinical significance of mucus plugging in airway diseases. Although pediatric MPP and cystic fibrosis exhibit distinct pathophysiological mechanisms, quantitative imaging techniques offer an objective morphological dimension for evaluating mucus plugs. Integrating BALF-derived molecular biomarkers with advanced imaging features through multimodal data fusion may represent a promising future strategy to improve BMP risk assessment and enhance individualized airway management.Several limitations of the present study should be acknowledged. First, owing to the single-center prospective design, the sample size was relatively limited and external independent cohort validation was lacking. Therefore, larger multicenter studies are still required to further evaluate the robustness and clinical generalizability of the predictive model. Second, the present study primarily explored the potential association between the TLR2/MyD88/MUC5AC signaling axis and bronchial mucus plug formation based on clinical specimens and statistical analyses. Although the results of the serial mediation analysis were consistent with the proposed mechanistic hypothesis, these findings remain exploratory and warrant further validation using airway epithelial cell models and animal experiments.

## Conclusion

The present study demonstrated that the association between TLR2 and BMP formation may involve a potential pathway including MyD88 and MUC5AC. Further analyses identified D-dimer, TLR2, and MUC5AC as independent risk factors for bronchial mucus plug formation. The nomogram model constructed based on these indicators showed favorable predictive performance and may facilitate identification of children at high risk of extensive bronchial mucus plug formation and support individualized airway management.

## Data Availability

The datasets generated and/or analyzed during the current study are not publicly available due to patient privacy and ethical restrictions but are available from the corresponding author on reasonable request.

## **Abbreviations**

ALT: Alanine aminotransferase
APTT: Activated partial thromboplastin time
AUC: Area under the curve
BALF: Bronchoalveolar lavage fluid
BMPs: Bronchial mucus plugs
CAP: Community-acquired pneumonia
CK-MB: Creatine kinase-MB
CRP: C-reactive protein
DCA: Decision curve analysis
ELISA: Enzyme-linked immunosorbent assay
INR: International normalized ratio
IQR: Interquartile range
IVIG: Intravenous immunoglobulin
LASSO: Least absolute shrinkage and selection operator
LDH: Lactate dehydrogenase
MPP: Mycoplasma pneumoniae pneumonia
MP: Mycoplasma pneumoniae
MUC5AC: Mucin 5AC
MyD88: Myeloid differentiation primary response 88
NF-κB: Nuclear factor kappa-B
IL-6: Interleukin-6
OR: Odds ratio
ROC: Receiver operating characteristic
CI: Confidence interval
SVM: Support vector machine
TLR2: Toll-like receptor 2
TNF-α: Tumor necrosis factor-alpha
TT: Thrombin time
PTA: Prothrombin activity
